# Longer duration of amenorrhea is associated with lower delay discounting and less hedonic eating in girls and young women with low-weight eating disorders

**DOI:** 10.1038/s41598-026-45493-2

**Published:** 2026-04-03

**Authors:** Marie-Louis Wronski, Franziska Plessow, Madeline Rogers, Meghan Slattery, Stefan Ehrlich, Daniel S. Quintana, Kjersti Mæhlum Walle, Lauren Breithaupt, Jennifer J. Thomas, Laura M. Holsen, Elizabeth A. Lawson, Madhusmita Misra, Kamryn T. Eddy

**Affiliations:** 1 Neuroendocrine Unit, Department of Medicine, Massachusetts General Hospital and Harvard Medical School, Boston, MA USA; 2 Translational Developmental Neuroscience Section, Division of Psychological and Social Medicine and Developmental Neurosciences, Faculty of Medicine, Technische Universität Dresden, Dresden, Germany; 3 Mass General Brigham Multidisciplinary Eating Disorders Research Collaborative, Mass General Brigham, Boston, MA USA; 4 Department of Psychiatry and Behavioral Sciences, Weill Institute for Neurosciences, University of California San Francisco, San Francisco, CA USA; 5 Eating Disorder Treatment and Research Center, Department of Child and Adolescent Psychiatry, Faculty of Medicine, University Hospital Carl Gustav Carus, Technische Universität Dresden, Dresden, Germany; 6 German Center for Child and Adolescent Health (DZKJ), Partner Site Dresden/Leipzig, Dresden, Germany; 7 Department of Psychology, University of Oslo, Oslo, Norway; 8 NevSom, Department of Rare Disorders and Disabilities, Oslo University Hospital, Oslo, Norway; 9 Eating Disorders Clinical and Research Program, Department of Psychiatry, Massachusetts General Hospital and Harvard Medical School, Boston, MA USA; 10 Division of Child and Adolescent Psychiatry, AMC Department of Psychiatry, Mass General Brigham and Harvard Medical School, Boston, MA USA; 11 Psychiatry Neuroimaging Laboratory, Department of Psychiatry, Brigham and Women’s Hospital and Harvard Medical School, Boston, MA USA; 12 Division of Women’s Health, Department of Medicine and Department of Psychiatry, Brigham and Women’s Hospital and Harvard Medical School, Boston, MA USA; 13 Division of Pediatric Endocrinology, Department of Pediatrics, University of Virginia School of Medicine, Charlottesville, VA USA

**Keywords:** Amenorrhea, Delay discounting, Menstrual status, Hedonic eating, Low-weight eating disorders, Reward sensitivity, Diseases, Endocrinology, Health care, Medical research, Psychology, Psychology

## Abstract

Oligo-amenorrhea is common in low-weight eating disorders (LWEDs) and reflects cumulative suppression of the hypothalamic-pituitary-gonadal axis, leading to chronic or cumulative hypoestrogenism. Estrogen modulates mesolimbic dopaminergic signaling involved in reward behavior. Investigating amenorrhea-reward mechanisms in LWEDs is clinically relevant, as reduced reward responsiveness and associated altered decision-making may sustain disordered eating. We examined whether lifetime duration of amenorrhea is associated with reward-based decision-making and hedonic eating in females with LWEDs. We hypothesized that longer amenorrhea duration would correlate with reduced discounting of delayed rewards and lower hedonic food intake. Fifty-six females with LWEDs (age median[IQR] = 19.8[3.0] years) and 34 female healthy controls (HCs; 19.1[2.4] years) completed assessment of menstrual history, a monetary Delay Discounting Task, and a fed-state Cookie Taste Test (task to evaluate hedonic eating). Between-group differences were tested using Mann-Whitney U-tests. In the LWED group, partial Spearman correlations, adjusted for age and body mass index (BMI), examined associations of amenorrhea duration with discounting rate *k* and hedonic food intake. Participants with LWEDs reported a median of 16.5(22.6) missed menses since menarche. In LWEDs, longer amenorrhea duration correlated with shallower discounting (lower [ln]*k*; *ρ* = -0.40, p = 0.004) and lower hedonic eating task caloric intake (*ρ* = -0.34, p = 0.011); discounting and hedonic intake were not intercorrelated. These findings suggest that greater cumulative periods of hypogonadism may impact monetary reward sensitivity and hedonic eating in LWEDs, independent of age and BMI-defined illness severity. Prospective interventional studies should test whether modifying gonadal hormone exposure can alter delay discounting and pleasurable eating in this population.

## Introduction

Low-weight eating disorders (LWEDs) such as anorexia nervosa (AN) are characterized by a high drive for thinness, a fear of weight gain, and a pattern of self-starvation^1^. They primarily affect young females with modal onset during adolescence, a time of critical brain development and maturation of the hypothalamic-pituitary-gonadal (HPG) axis^2^. Alterations in the reward system have been documented in LWEDs and may contribute to treatment resistance and poor clinical outcomes in this population^3^. Delay discounting—the devaluation of future rewards with increased delay to receipt^4–6^—is commonly reduced in individuals with LWEDs who tend to more strongly favor larger, delayed over smaller, more immediate rewards^7,8^. However, evidence on delay discounting in LWEDs is inconsistent. Several studies have reported no significant differences in delay discounting between individuals with acute and recovered LWEDs, particularly AN, and controls^7,9–12^. These findings underscore the heterogeneity of delay discounting results in eating disorders and highlight the need for further systematic investigation. Additionally, the hedonic consumption of palatable food, also suggestive of reward responsiveness and mediated by the brain’s dopaminergic reward system^13^, is typically reduced in individuals with LWEDs^14^. Nevertheless, despite the limited evidence base, one study reported that perceptual hedonic measures, such as hedonic ratings of specific tastes, did not differ between adolescents with AN and healthy controls^15^. While the biological mechanisms maintaining restrictive eating in LWEDs remain incompletely understood, enhancing reward responsiveness and altering reward-based decision-making may be critical for developing novel, targeted interventions for this population^3^.

LWEDs are characterized by significant neuroendocrine disruptions^16^, including suppression of the HPG axis leading to gonadal hormone alterations, notably estrogen deficiency^17^. Estrogen deficiency clinically manifests as infrequent menstrual periods (oligomenorrhea), the absence of menstruation by age 15 years (primary amenorrhea), and/or the cessation of menstrual periods after menarche (secondary amenorrhea)^18^. Estrogen receptors are widely expressed in the brain, including in the ventral striatum and the amygdala, both of which are central nodes of the mesolimbic dopaminergic reward pathway^19^. This neural pathway is involved in reward-driven decision-making and behaviors, including delay discounting and hedonic eating^20^. Estrogen is known to affect dopamine metabolism in the brain, indirectly acting as an endogenous dopamine agonist^19,21^, which suggests that estrogen exposure may play a role in modulating reward processing. Although the evidence base is comparatively limited, progesterone, the other major female gonadal hormone, appears to act mainly as a modulatory counterpart to estrogen, potentially dampening its facilitative effects on dopaminergic reward processing^22^.

Links between menstrual status and reward sensitivity, food intake, and social behavior have been demonstrated in both animal models and preliminary human studies^23^, but not yet in LWED populations. For instance, healthy female adults demonstrated reduced delay discounting after a rise in estrogen levels in the mid-follicular phase as compared to the menstrual phase^24^. In contrast, decreasing estrogen levels in healthy women after administering a gonadotropin-releasing hormone agonist led to a reduction of the amygdala’s response to high monetary rewards^25^. Administering a combination of estrogen and progesterone preparations to perimenopausal women, in turn, enhanced reward-related striatal reactivity^26^. In early postmenopausal women, hormone therapy with estrogen and progesterone improved cognitive flexibility and augmented dorsolateral prefrontal cortex activation^27^. Functional hypothalamic amenorrhea, a condition triggered by weight loss, insufficient caloric intake, excessive exercise, and/or psychological distress, is characterized by suppressed hypothalamic activity, which in turn reduces gonadotropin-releasing hormone secretion and leads to low gonadal hormone levels^28^. Cognitive dysfunction^29^ and more severe depressive and anxiety symptoms^30–32^ have been observed in individuals with functional hypothalamic amenorrhea, such as normal-weight oligo-amenorrheic athletes^31^.

Estrogen supplementation is already a well-established therapy for various conditions, including estrogen deficiency during/after the menopausal transition and its metabolic and dermatological sequelae^26,27,33^. Similarly, while weight regain remains the primary goal, physiologic estrogen supplementation, such as transdermal delivery of 17β-estradiol (transdermal application helps protect bone health; combined with cyclic oral progesterone^34^), may offer benefits for young females with LWEDs who are in a hypoestrogenic state. Transdermal estrogen replacement (combined with cyclic oral progesterone) has been shown to reduce trait anxiety in girls with AN^35^ and to lessen drive for thinness and body dissatisfaction in normal-weight, oligo-amenorrheic athletes^36^. However, the strength and direction of a potential association between altered gonadal hormone exposure and reward sensitivity in LWEDs remain unclear.

In this study, we investigated the relationships between lifetime disruptions in gonadal hormone exposure, characterized by the cumulative duration of amenorrhea since menarche, and monetary delay discounting and hedonic food intake during a standardized fed-state hedonic eating task in girls and young women with LWEDs. Together, both behavioral measures capture decision-value and consummatory facets of reward most relevant to LWEDs in this developmental window. We hypothesized that a longer duration of amenorrhea would be associated with reduced delay discounting in an established monetary Delay Discounting Task^6^ (Hypothesis 1). In addition, we anticipated that a longer duration of amenorrhea would correlate with lower reward-related food intake in a hedonic eating task^37^ (Hypothesis 2).

## Materials & methods

### Study participants

The study sample included 56 female participants with LWEDs (aged 13.6–23.5 years) and 34 female healthy controls (HCs, aged 13.4–22.2 years). Participants were recruited from a prospective study that followed individuals with LWEDs and HCs over 18 months to study the neurobiology underlying LWED trajectories^38–40^. With a sample of 56 participants, a test evaluating the relationship between the duration of amenorrhea and test performance can reliably detect (i.e., with 80% power) an effect size of *ρ* = 0.36, or larger, with α = 0.05 (two-tailed).

HCs were selectively recruited to match participants with LWEDs for age (within 2 years) and Tanner stage (within 1 stage). HCs were required to have a normal body weight (body mass index [BMI] between 25th and 85th percentiles), menstruate regularly, have no pubertal delay, no lifetime history of psychiatric disorders, including EDs, and no use of psychoactive medications. In the LWED group, the presence of a LWED was characterized by (i) low body weight (defined as ≤ 90% of expected body weight based on the 50^th^ percentile BMI-for-age according to Centers for Disease Control and Prevention growth charts^41^) and (ii) restrictive eating, and/or bingeing, purging, and/or excessive exercise more than once per month, and/or treatment for an ED as assessed by the Eating Disorder Examination (EDE) clinical interview according to DSM-5 criteria^1^. The LWED sample comprised individuals diagnosed with either AN or atypical AN, including both restricting and binge-eating/purging types. Note that, although some of them were low-weight and amenorrheic^42^, individuals with avoidant/restrictive food intake disorder (ARFID, included in the parent study) were excluded from this investigation, given emerging evidence for ARFID’s distinct neurocognitive profile from AN and atypical AN, which are the focus of this study^43^. The following exclusion criteria applied to all participants: premenarcheal state; current exogenous estrogen or progesterone (e.g., hormonal birth control) or systemic hormone treatment; pregnancy or breast feeding within eight weeks prior to study participation; a history of psychosis or substance/alcohol use disorder as assessed by the Schedule for Affective Disorders and Schizophrenia for School-Aged Children Present and Lifetime Version (K-SADS-PL)^44^; active suicidal ideation; diabetes mellitus, anemia, or low body weight caused by medical conditions other than EDs. According to our standardized medical history assessment and screening laboratory tests, none of the participants reported any ED-unrelated causes of amenorrhea (e.g., pregnancy, hypothalamic or pituitary inflammation, trauma, or tumor, hyperprolactinemia, thyroid dysfunction, polycystic ovary syndrome, primary ovarian insufficiency).

The study was approved by the Institutional Review Board of Mass General Brigham (MGB) and conducted in accordance with the Declaration of Helsinki. Written informed consent was obtained from all participants ≥ 18 years, or assent from participants and written informed consent from legal guardians if participants were < 18 years old.

### Screening assessments

The eligibility of study participants, including review of their medical history, physical examination, safety blood and urine laboratory assessments, clinical interviews, and demographic characteristics, such as race and ethnicity, were assessed at a screening visit. ED-related psychopathology was captured with the global score of the EDE clinical interview^45^. All research data were managed using a secure, web-based data capture tool (REDCap)^46^.

### Menstrual history

As per inclusion criteria, all participants in the study sample were postmenarcheal. A detailed menstrual history assessment was conducted at the main study visit by a trained study physician or nurse practitioner. This assessment included age at menarche and standardized assessment (by recall) of the number of missed menses since menarche using customized time intervals based on each participant’s current age and age at menarche. For instance, participants were asked how many periods they had per year between (i) menarche and age 15 years, (ii) 15 to 18 years old, and (iii) 18 to 22 years old. The number of missed menses was then calculated, assuming an average of twelve periods per year. Total lifetime duration of (secondary) amenorrhea was estimated as the cumulative number of missed menstrual cycles since menarche.

### Food and fluid intake during the study visit

**Fig. 1:**
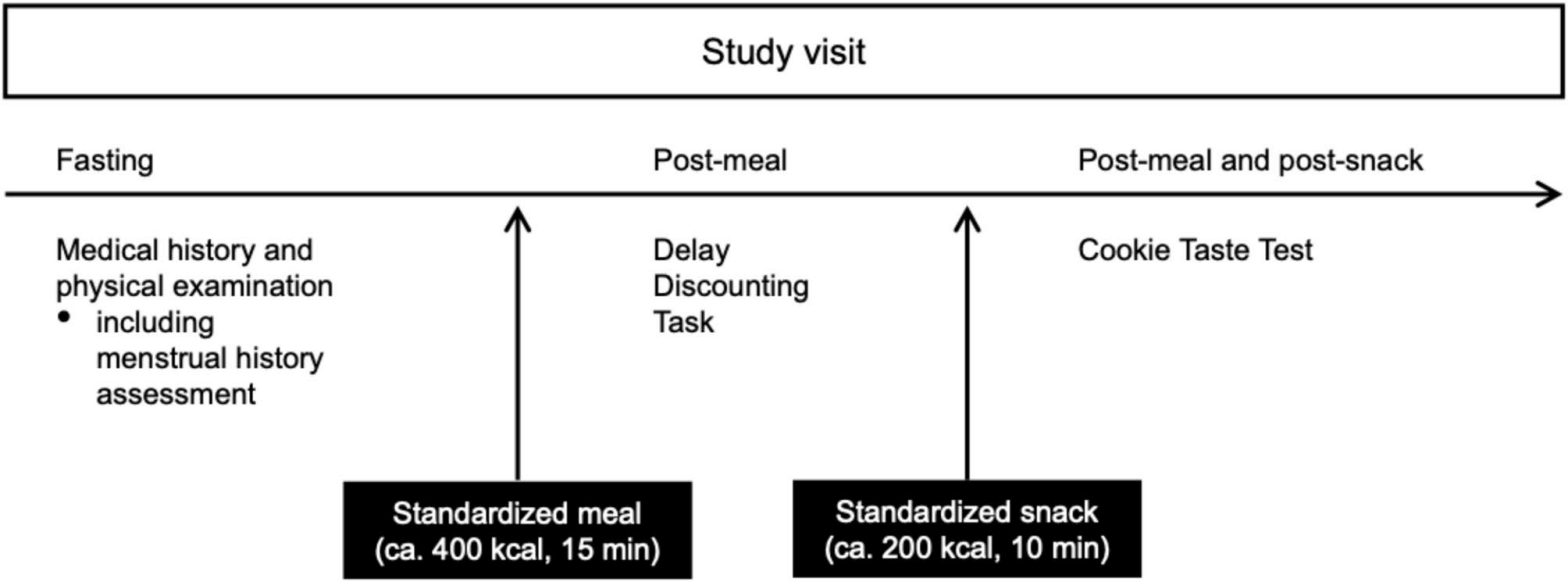
Procedures during the study visit.

At the main study visit, following medical, including menstrual history assessment and physical examination, participants consumed a standardized mixed-meal breakfast (~ 400 kcal). After completion of subsequent study procedures (approximately 150 min post-breakfast), participants were presented with a standardized snack (~ 200 kcal; Fig. 1). Participants selected from three predefined breakfast options and three predefined snack options. Breakfast options included: (1) milk, yogurt, and cereal; (2) mini bagel, peanut butter, lactose-free milk, and dried sweetened cranberries; and (3) gluten-free toast, lactose-free milk, yogurt, peanut butter, and dried sweetened cranberries. Snack options included: (1) potato chips and yogurt; (2) gluten-free toast with butter and yogurt; and (3) applesauce, peanut butter crackers, and lactose-free milk.

Breakfast and snack options were standardized with respect to macronutrient composition (breakfast: ~ 60% carbohydrate, ~ 20% fat, ~ 20% protein; snack: ~ 80% carbohydrate, ~ 10% fat, ~ 10% protein). Meals were individualized to accommodate participants’ dietary requirements and reported food allergies or intolerances; however, macronutrient content was held constant to ensure comparability across participants. The mixed meal was used in our prior work examining food motivation pathways in AN, in which individuals with AN and healthy controls did not differ in the nutritional content of the meals consumed^47–49^.

Regarding fluid intake, breakfast included a cow- or soy-based milk drink. Outside meals and snacks, water was available ad libitum.

Daily caloric intake, derived from 24-h dietary recalls, was used as a reference measure to compute the percentages of daily intake consumed during the standardized mixed-meal breakfast, the snack, and the Cookie Taste Test. Food recalls were conducted in person at the Translational and Clinical Research Center (TCRC) by trained registered dietitians and dietetic technicians using a standardized multiple-pass interview approach comprising four passes (collection of a quick list, review of the quick list, collection of detailed recall information, and review of detailed recall information). Participants were systematically prompted to report all foods, beverages, snacks, seasonings, condiments, and dietary supplements consumed during the previous 24 h. For each reported item, detailed information was collected on food type, brand or product name, preparation method, specific cuts or varieties, and any ingredients added or removed during preparation or prior to consumption. Portion sizes were estimated using standardized measurement units (e.g., ounces, fluid ounces, cups, teaspoons, and tablespoons) and supported by visual portion-size aids, including food models and images of cups, plates, and glasses, to enhance accuracy.

Intake data were entered directly into the Nutrition Data System for Research (NDSR), a computer-based dietary analysis software developed by the Nutrition Coordinating Center (NCC) at the University of Minnesota (Minneapolis, MN, USA)^50,51^. The NDSR software generates nutrient reports in total and per food group from entered recall data and is based on food composition data from the NCC Food and Nutrient Database, which relies on the U.S. Department of Agriculture National Nutrient Database^52^.

Quality control procedures included intensive staff training in standardized nutrient collection and analysis, as well as independent review of all recall data and NDSR-generated nutrient reports for completeness and accuracy by the primary registered dietician for the study, who implemented necessary error corrections and excluded clearly artefactual data. Subsequently, validated food recall data were exported to spreadsheets and routinely backed up.

### Monetary delay discounting task

The monetary Delay Discounting Task, a computerized version of Kirby’s paper-and-pencil Monetary Choice Questionnaire^4,5^, implemented using Presentation® software (Neurobehavioral Systems Inc./Berkeley/CA/USA), was performed at the main study visit, approximately 60 min after participants had the mixed-meal breakfast (Fig. 1). Participants were asked to make a total of 27 choices between smaller, immediate monetary rewards and larger, delayed monetary rewards (e.g., “$10 today or $50 in 7 days”). Based on participants’ choices, an overall delay discounting rate *k* was computed as an index of impulsive decision-making, derived by estimating *k* for each of the 27 trials (formula: V = A/[1 + *k*D], where V = subjective value of the delayed reward, A = actual amount of the delayed reward, *k* = discounting rate, and D = time delay) and aggregating the results^6^. The parameter *k* was then log_e_(*ln*)-transformed to approximate a normal distribution. Smaller *k* values reflect shallower delay discounting, resulting in a stronger preference for larger, delayed over smaller, more immediate rewards.

Discounting rates *k* were computed overall (27 trials, see above) and separately for each of the three reward magnitude categories (small, medium, large; 9 trials each; see Table 1 for details)^4–6^. Additionally, the proportion of larger, delayed reward choices (LDR), which refers to the proportion of times participants opted for the larger, delayed reward option over the immediate reward option, was determined both in total and by reward magnitude category. LDR served as a complementary, bounded index of the participants’ willingness to wait for a larger reward, supporting magnitude-specific sensitivity checks and cross-study comparability^53^.

**Table 1:** Descriptive statistics of the study sample.

Variable	LWED (n = 56)	HC (n = 34)	U statistic	p	Wilcoxon r
Age (years)	19.84 (2.98)	19.08 (2.41)	791.50	0.183	0.14
BMI (kg/m^2^)	17.97 (2.16)	21.71 (2.57)	1,802.50	< 0.001	0.75
BMI z score (age < 20 years: n = 30 LWED, n = 22 HC)	-1.22 (0.98)	0.07 (0.85)	641.00	< 0.001	0.61
EDE global score	2.38 (2.50)	0.00 (0.00)	22.50	< 0.001	0.83
Daily caloric intake derived from 24-h recall^**a**^	2,158.33 (1,422.53)	1,638.49 (806.07)	1,001.00	0.036	0.23
VAS hunger fasting/pre-meal^**b**^	52.00 (40.00)	65.00 (16.50)	1,248.00	0.008	0.28
Standardized meal caloric intake (kcal)	401.81 (54.37)	401.81 (54.83)	1,016.50	0.593	0.06
- % of daily caloric intake	17.11 (12.89)	22.14 (11.13)	572.00	0.041	0.22
Standardized snack caloric intake (kcal)	204.03 (38.36)	210.81 (19.56)	1,066.00	0.342	0.10
- % of daily caloric intake	9.27 (6.28)	11.65 (6.50)	557.00	0.029	0.23
***Race***					
- Asian	9 (16.07%)	6 (17.65%)		> 0.999	
- White	46 (82.14%)	28 (82.35%)			
- Other	1 (1.79%)				
***Ethnicity***					
- Hispanic or Latino	4 (7.14%)	1 (2.94%)		0.646	
- Not Hispanic or Latino	52 (92.86%)	33 (97.06%)			
Duration of illness (months)^**c**^	18.06 (28.66)	n/a	n/a	n/a	n/a
*LWED diagnoses*		n/a	n/a	n/a	n/a
- Restricting AN	19 (33.93%)				
- Binge-eating/purging AN	10 (17.86%)				
- Atypical restricting AN	21 (37.50%)				
- Atypical binge-eating/ purging AN	6 (10.71%)				
*Co-existing psychiatric diagnoses*		n/a	n/a	n/a	n/a
- Depressive disorder	9 (16.07%)				
- Generalized anxiety	28 (50.00%)				
- Panic disorder	9 (16.07%)				
- Social anxiety	11 (19.64%)				
- Separation anxiety	2 (3.57%)				
- Phobic disorder	5 (8.93%)				
- Obsessive–compulsive disorder	2 (3.57%)				
- Post-traumatic stress disorder	2 (3.57%)				
- Attention-deficit/hyperactivity disorder	2 (3.57%)				
*Psychoactive medications*		n/a	n/a	n/a	n/a
- Selective serotonin reuptake inhibitors	19 (33.93%)				
- Serotonin-norepinephrine reuptake inhibitors	3 (5.36%)				
- Mirtazapine	1 (1.79%)				
- Benzodiazepines	5 (8.93%)				
- Stimulants	2 (3.57%)				
- Mood stabilizers	2 (3.57%)				
- Antipsychotic medications	3 (5.36%)				
- Antihistamines	1 (1.79%)				
*Current treatment programs*		n/a	n/a	n/a	n/a
- Residential treatment centers	14 (25.00%)				
- Full-day treatment programs	3 (5.36%)				
- Intensive outpatient programs	2 (3.57%)				
- Outpatients	25 (44.64%)				
- No treatment	12 (21.43%)				
Total lifetime duration of amenorrhea (since menarche, months)	16.50 (22.64)	n/a	n/a	n/a	n/a
**Delay discounting task**					
Ln(*k*) overall^**d**^	-5.99 (3.19)	-5.76 (1.53)	933.50	0.273	0.12
Ln(*k*) small	-5.56 (2.29)	-4.63 (1.18)	964.50	0.164	0.15
Ln(*k*) medium	-6.45 (3.08)	-5.54 (2.73)	930.50	0.283	0.11
Ln(*k*) large	-6.44 (3.21)	-6.44 (2.04)	954.50	0.192	0.14
LDR overall (%)	66.67 (35.19)	55.56 (23.15)	681.50	0.209	0.13
LDR small (%)	55.56 (27.78)	44.44 (16.67)	709.00	0.312	0.11
LDR medium (%)	66.67 (38.89)	55.56 (33.33)	701.00	0.279	0.11
LDR large (%)	66.67 (38.89)	66.67 (25.00)	673.50	0.179	0.14
Consistency (%)	96.30 (7.41)	92.59 (3.70)	690.50	0.224	0.13
**Cookie Taste Test**					
VAS hunger pre-Cookie Taste Test^**e**^	16.00 (35.50)	24.00 (24.00)	1,097.00	0.142	0.15
Total intake (g)	15.00 (16.03)	34.65 (43.98)	1,559.50	< 0.001	0.53
Caloric intake (kcal)^**f**^	64.47 (73.52)	153.72 (136.26)	1,544.00	< 0.001	0.52
- % of daily caloric intake	3.61 (4.01)	8.13 (12.36)	301.00	< 0.001	0.49

*AN, anorexia nervosa; BMI, body mass index; EDE, Eating Disorder Examination (clinical interview); HC, healthy controls; LDR, larger, delayed reward; ln(k), natural logarithm (base e) of delay discounting rate k; LWED, low-weight eating disorder; VAS, visual analog scale.*

Data distributions were visually inspected and formally tested using Shapiro–Wilk test (normality assumption) and Levene test (variance homogeneity assumption). For study groups LWED and HC, n (%) are stated for count variables. Median (interquartile range) are stated for continuous variables. Given deviations from normality in several variables, group differences between LWED and HC were tested using nonparametric Mann Whitney U-test (U-value, p-value, and effect size estimate (Wilcoxon’s r) are stated; Fisher’s exact test was applied for categorical/count variables). Delay Discounting task characteristics are provided overall and for different reward magnitudes (small: LDR amounts $25, $30, and $35; medium: LDR amounts $50, $55, and $60; large: LDR amounts $75, $80, and $85). ^**a**^Daily caloric intake was estimated using a 24-h dietary recall completed on the day preceding the main study visit. ^**b**^VAS *“How hungry do you feel?”* rating (scale: 0–100) assessed in the fasting state (pre-meal), serving as a reference. ^**c**^Months since clinical diagnosis. ^**d**^LWED diagnostic subgroups did not differ in ln(*k*) overall. Ln(*k*) overall median (IQR) in restricting AN: -6.45 (2.72), binge-eating/purging AN: -5.77 (2.21), atypical restricting AN: -6.22 (2.97), and atypical binge-eating/purging AN: -6.22 (4.61). Kruskal–Wallis ANOVA for ln(*k*) overall differences between LWED subgroups: H = 0.17, p = 0.983. ^**e**^VAS *“How hungry do you feel?”* rating (scale: 0–100) assessed in the postprandial state, prior to the Cookie Taste Test. ^**f**^LWED diagnostic subgroups did not differ in Cookie Taste Test caloric intake. Caloric intake median (IQR) in restricting AN: 56.58 (93.98) kcal, binge-eating/purging AN: 73.79 (53.29) kcal, atypical restricting AN: 67.47 (69.79) kcal, and atypical binge-eating/purging AN: 87.36 (77.22) kcal. Kruskal–Wallis ANOVA for differences in caloric intake between LWED subgroups: H = 0.43, p = 0.934.

### Cookie taste test

The Cookie Taste Test (adapted from a similar behavioral task^37^ and henceforth referred to as hedonic eating task) was performed approximately 180 min after the mixed-meal breakfast and 30 min after the snack in order to capture hedonic eating in a fed state (Fig. 1)^14^. Subjective hunger was assessed via Visual Analog Scale (VAS) ratings in the fasting (pre-meal, used as a reference) and fed (post-meal) states to confirm that homeostatic hunger was low prior to performing the hedonic eating task. During the hedonic eating task, participants were asked to eat as much of three different types of cookies, matched for macronutrient content, as they desired and needed to rate the taste of the cookies. Different cookie types were offered to provide flavor options for individual preferences and meet individual dietary requirements. The amount (weight) of cookies consumed was then measured to compute caloric intake using the ProNutra™ software (VioCare Inc./Princeton/NJ/USA).

### Statistical analyses

Statistical analyses were performed in R version 4.1.1 (R Core Team 2022/Vienna/Austria). Following common quality-control practices in intertemporal choice research, three participants in the study sample were excluded from the Delay Discounting Task analysis (but included in all other analyses): one HC exhibited response times < 250 ms, suggestive of anticipatory responses too fast for considerate decision-making^54^; one individual with a LWED and one HC showed overall choice consistency < 75%, indicating low attention to the task^55^.

Data distribution was visually inspected using boxplots and formally tested using Shapiro–Wilk test (normality assumption) and Levene test (variance homogeneity assumption). Given deviations from normality, differences between LWED and HC groups in continuous variables were tested with the nonparametric Mann Whitney U-test. Group differences in categorical/count variables (race, ethnicity) were tested with Fisher’s exact test. An α level of 0.05 (two-tailed) was considered statistically significant.

**Fig. 2:**
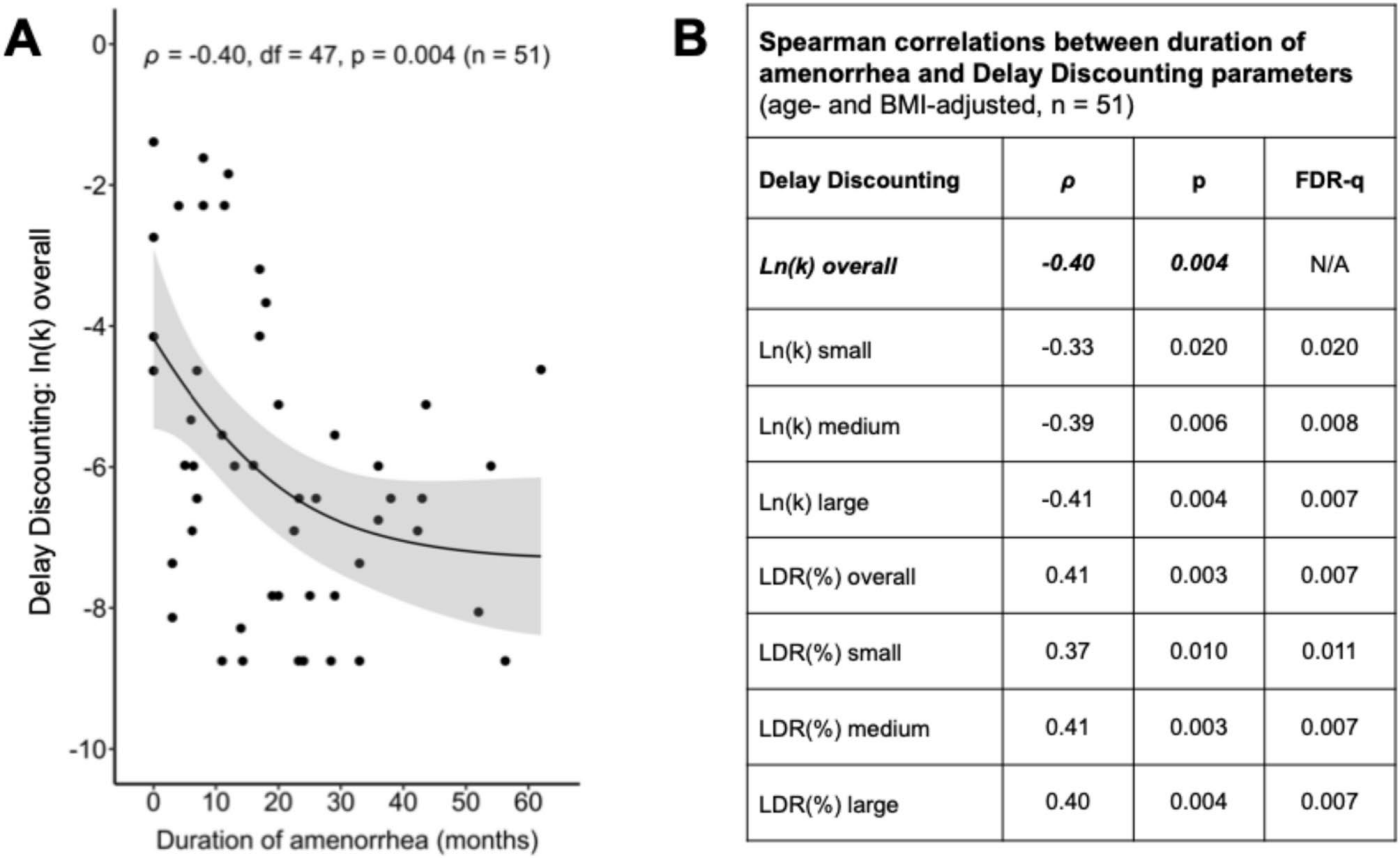
Association between duration of amenorrhea since menarche and Delay Discounting Task performance in individuals with low-weight eating disorders (LWEDs). *LDR, larger, delayed reward; ln(k), natural logarithm (base e) of delay discounting rate k. ***(A):** Scatter plot displaying the association between duration of amenorrhea since menarche and natural log-transformed delay discounting rate *k* in participants with LWEDs. A monotonic curve and 95% confidence interval were fitted to the raw (unadjusted) data. Spearman correlation statistics, adjusted for age and BMI, are stated. **(B):** Table of Spearman correlations (partial, adjusted for age and BMI) between duration of amenorrhea since menarche and delay discounting parameters (overall and for individual reward magnitudes: small: LDR amounts $25, $30, and $35; medium: LDR amounts $50, $55, and $60; large: LDR amounts $75, $80, and $85) in participants with LWEDs. Spearman correlation coefficient (*ρ*), p-value, and multiple testing-adjusted p-value (FDR-q) are stated. False discovery rate (FDR) adjustment was applied across all assessed correlations except for ln(*k*) overall (main hypothesis).

Within the LWED group, associations between duration of amenorrhea since menarche and (i) delay discounting parameter *k* and proportion of larger, delayed rewards chosen, as well as (ii) caloric intake during the hedonic eating task were examined via partial Spearman rank-order correlations. Correlations were adjusted for age and BMI to control for potential confounding effects of age and the severity of underweight on the examined associations. Multiple testing correction was applied using the Benjamini–Hochberg false discovery rate^56^ procedure across all assessed correlations between amenorrhea duration and reward measures (e.g., *k* and the proportion of larger, delayed rewards chosen across reward magnitude categories, n = 7 tests [Fig. 2B]), with the exception of correlations involving overall *k* and hedonic eating task caloric intake, which represented the study’s primary hypotheses (n = 2 tests). Sensitivity analyses comprised adjusting the correlation between amenorrhea duration and (i) overall *k* for illness duration, (ii) overall *k* for eating disorder symptom severity (EDE global score), (iii) hedonic eating task caloric intake for illness duration, (iv) hedonic eating task caloric intake for EDE global score, and (v) hedonic eating task caloric intake for total caloric intake during the study visit preceding the hedonic eating task (i.e., caloric intake at the standardized meal plus the snack), in addition to adjustment for age and BMI.

As a follow-up analysis, contingent on meeting statistical assumptions, we planned to test whether the relationship between duration of amenorrhea and hedonic eating during the hedonic eating task was mediated by the impact of amenorrhea duration on reward-based decision-making in the Delay Discounting task using regression-based mediation analysis.

## Results

### Descriptive statistics

LWED and HC study groups did not differ in age (overall median [IQR] = 19.5 [2.8] years), race, and ethnicity (Table 1). As per study design, participants with LWEDs had lower BMI (18.0 [2.2] kg/m^2^) than HCs (21.7 [2.6] kg/m^2^) and presented with more pronounced ED symptoms than HCs (Table 1). While VAS homeostatic hunger ratings were higher in HCs than LWEDs in the fasting state, absolute caloric intake during the standardized mixed-meal breakfast and the snack did not differ between LWED and HC groups (meal kcal range LWED: 50.8–427.9 kcal, HC: 103.1–427.9 kcal; snack kcal range LWED: 0–277.0 kcal, HC: 49.2–250.7 kcal; see Table 1 for median and interquartile range summary statistics). Self-reported 24-h caloric intake was higher and more variable in LWED than in HC participants; accordingly, the proportion of daily intake consumed during the meal and snack differed between groups (Table 1).

The median duration of secondary amenorrhea was 16.5 months in participants with LWEDs (assessed in all 56 LWED participants; range [min–max] = 0–62 months). In accordance with the inclusion criteria, secondary amenorrhea did not occur in HCs. We did not observe significant differences in delay discounting rates overall or for individual reward magnitudes between the LWED group (ln[*k*] overall = -6.0 [3.2]) and the HC group (-5.8 [1.5]). Participants with LWEDs did not select a statistically significantly higher percentage of larger, delayed rewards (66.7 [35.2] %) than HCs (55.6 [23.2] %). VAS ratings indicated that homeostatic hunger was low and comparable between the LWED and HC groups prior to the hedonic eating task, both in absolute terms and relative to the fasting state (Table 1). Participants with LWEDs consumed significantly fewer calories (64.5 [73.5] kcal) than HCs (153.7 [136.3] kcal) during the hedonic eating task. Supplementary analyses revealed that individuals with LWEDs who were primarily restricting and those who were additionally engaging in binge-eating/purging showed a similar delay discounting rate and did not differ in calories consumed during the hedonic eating task (see Table 1 legend). Additional demographic and clinical details for LWED participants are provided in Table 1, such as duration of illness, co-existing psychiatric diagnoses, psychoactive medications, and current treatment programs.

### Duration of lifetime amenorrhea and delay discounting

Among participants with LWEDs, a longer duration of amenorrhea since menarche was significantly associated with a lower discounting rate *k* (*ρ* = -0.40, p = 0.004), consistent with shallower delay discounting (Fig. 2A). This inverse association between amenorrhea duration and *k* overall remained significant after adjusting for (i) the duration of illness since clinical diagnosis (*ρ* = -0.32, p = 0.041), as well as (ii) EDE global score (*ρ* = -0.41, p = 0.004) in addition to the standard covariates age and BMI. Furthermore, inverse associations of similar strength between amenorrhea duration and discounting rates *k* were observed across reward magnitudes (i.e., small, medium, and large rewards; Fig. 2B). Mirroring these findings, longer amenorrhea duration in participants with LWEDs was associated with a higher percentage of larger, delayed reward choices, indicating a stronger preference for larger, delayed relative to smaller, immediate rewards (*ρ* = 0.41, p = 0.003). This association was consistent across reward magnitude levels, as well (Fig. 2B). All reported correlation results were independent of participant age and degree of underweight, as measured by BMI.

### Duration of lifetime amenorrhea and hedonic food intake

**Fig. 3:**
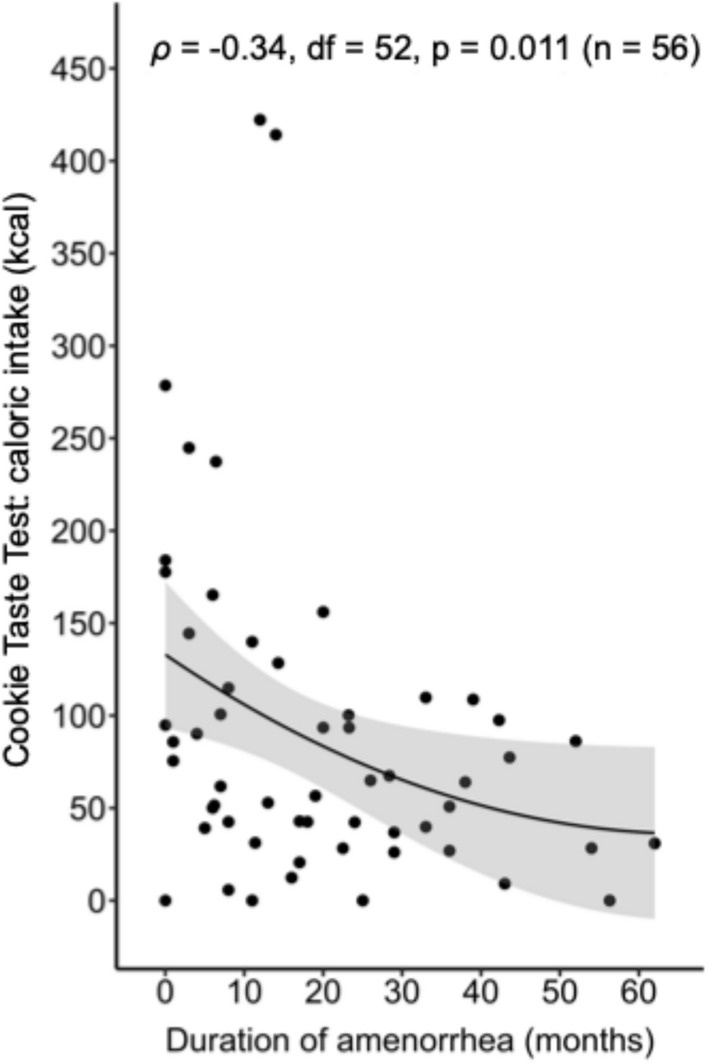
Association between duration of amenorrhea since menarche and caloric intake during the Cookie Taste Test in individuals with low-weight eating disorders (LWEDs). Scatter plot displaying the association between duration of amenorrhea since menarche and caloric intake during the Cookie Taste Test in participants with LWEDs. A monotonic curve and 95% confidence interval were fitted to the raw (unadjusted) data. Spearman correlation statistics, adjusted for age and BMI, are stated.

A longer duration of amenorrhea since menarche was associated with lower caloric intake during the hedonic eating task (*ρ* = -0.34, p = 0.011) in participants with LWEDs, independent of participant age and BMI (Fig. 3). This inverse correlation between amenorrhea duration and hedonic eating task caloric intake remained significant after adjusting for (i) illness duration since clinical diagnosis (*ρ* = -0.34, p = 0.024), (ii) EDE global score (*ρ* = -0.32, p = 0.020), as well as (iii) total caloric intake during the standardized meal and the snack prior to the hedonic eating task (*ρ* = -0.35, p = 0.011) in addition to the standard covariates age and BMI.

Follow-up Spearman correlation analysis within the LWED group revealed no significant association between hedonic eating task caloric intake and delay discounting rate *k* (*ρ* = 0.05, p = 0.731). Consequently, as the statistical assumptions for mediation were not met, we did not proceed with the planned mediation analysis examining whether the relationship between duration of amenorrhea and hedonic eating in participants with LWEDs was mediated by the effect of amenorrhea duration on reward-related decision-making (delay discounting).

## Discussion

To our knowledge, this is the first study to examine associations between lifetime gonadal hormone deficiency—indexed by the cumulative duration of (secondary) amenorrhea after menarche—and delay discounting and palatable eating in females with LWEDs. We found that longer amenorrhea duration was associated with shallower discounting (greater willingness to wait for delayed rewards) in a Delay Discounting Task and decreased hedonic food intake during a hedonic eating task. These associations were independent of participants’ age, degree of underweight (BMI), illness duration, eating disorder symptom severity, and caloric intake preceding the hedonic eating task. These findings suggest that reduced gonadal hormone exposure in individuals with LWEDs may modulate reward sensitivity beyond the effects of nutritional status alone, highlighting the potential clinical relevance of estrogen-(and progesterone-)based treatments in this population.

We did not observe group-level differences in delay discounting parameters between participants with LWEDs and HCs. Previous studies have yielded mixed findings^7–10,57,58^. However, an enhanced preference to delay rewards has frequently been observed in individuals with LWEDs, especially those with restricting type presentations^7,8,57^. This divergence from our finding may be attributable to factors such as heterogeneity of study populations regarding LWED presentations (e.g., typical vs. atypical AN, restricting vs. binge-eating/purging subtypes) or participants’ age and LWED chronicity, since the aforementioned studies included individuals who were, on average, older (mid-to-late twenties)^7,57^ and had a longer illness history (e.g., illness duration of five or more years^7^) relative to the present sample. Consistent with our findings, other studies involving younger, primarily adolescent LWED participants with a shorter illness duration—comparable to those in the present sample—have reported no group differences in delay discounting parameters between LWEDs and HCs^9,10,58^. Additionally, the LWED sample in this study was heterogeneous, comprising those with AN and atypical AN, and those with both restricting and binge-eating/purging type illnesses. Evidence suggests that individuals with restricting AN tend to exhibit reduced delay discounting, while those with binge-eating/purging AN may show unchanged^57^ or even elevated^7^ discounting rates. In our sample, an exploratory follow-up analysis did not reveal a difference in delay discounting rate *k* between those with restricting and those with binge-eating/purging type LWED presentations.

Hedonic appetite was altered in LWED participants, reflected by significantly lower caloric intake during the hedonic eating task in a fed state compared to HCs. We ensured, using VAS ratings, that the hedonic eating task captured hedonic rather than homeostatic eating, as subjective hunger was low in both study groups prior to the task. Our finding of lower hedonic food consumption in individuals with LWEDs than HCs is consistent with the literature^14^ and likely mirrors reduced reward-driven eating behavior in individuals with LWEDs, who may perceive palatable food cues as aversive rather than pleasurable^3^. In support, attenuated state and trait activation in dopaminergic reward-related brain regions, such as the amygdala and insula, has been demonstrated in acutely underweight and weight-restored adult females with AN^47^. However, in a separate study using the same sample, we found hyperactivation in neural regions, implicated in reward-memory *and* cognitive control processes, in those with LWEDs compared to HCs during a passive food cue viewing task^38^. This evidence raises the question of whether it is not simply an alteration in food-related reward responsiveness but also one of increased cognitive control that underlies reduced hedonic food intake in the LWED population. Consequently, cognitive overcontrol may lead to avoidance of pleasurable foods in individuals with LWEDs, further exacerbating disordered eating behaviors^59^.

In line with our hypotheses, longer duration of secondary amenorrhea, a proxy of cumulative gonadal hormone deficiency, was associated with both attenuated delay discounting (reflecting diminished reward-based decision-making) and reduced intake of palatable foods in a fed state (reflecting blunted hedonic eating) in individuals with LWEDs. Notably, the associations between amenorrhea duration and reward measures proved robust—remaining independent of age and severity of underweight (BMI) and not substantially altered by adjustment for the duration of illness, severity of eating disorder symptoms (EDE global score), and preceding food intake. However, delay discounting and hedonic eating were not linked in our study. Thus, we propose that gonadal hormone deficiency in LWEDs may exert independent effects on delayed gratification and hedonic eating pathways. Alternatively, a larger study sample may be necessary to demonstrate a mediation effect. To date, there are no prior studies in LWED populations allowing direct comparison with our findings. Nevertheless, supporting evidence comes from studies in individuals with functional hypothalamic amenorrhea, where increased cognitive, depressive, and anxiety symptoms have been documented^30–32^. Additionally, experimentally lowering estrogen levels resulted in diminished responsiveness to monetary rewards in an interventional study in healthy women^25^, which aligns with the directionality of our results. Further substantiating the directionality of our findings in LWEDs, studies in perimenopausal and early postmenopausal women demonstrated that combined estrogen and progesterone therapy enhanced reward sensitivity^26^ and cognitive flexibility^27^. Moreover, experimental estrogen treatment (combined with cyclic progesterone) in a clinical trial improved ED symptoms in athletes with oligo- or amenorrhea^36^. However, findings from human studies on the effects of estrogen exposure on reward sensitivity are not uniform. An inverted U-shaped relationship between estrogen concentration and reward sensitivity has been reported, with women exhibiting reduced reward sensitivity during the high-estrogen late follicular phase compared with cycle onset^21,60^.

In contrast—and counter to our hypotheses as well as the direction of the amenorrhea-reward sensitivity relationships observed in the present study—preclinical evidence indicates that estradiol reduces food intake and food-motivated behavior in female mammals. Across ovariectomy and estradiol-replacement paradigms, estradiol has been shown to decrease meal size, operant responding, and willingness to work for palatable food^61–63^. Additional rodent studies suggest that estradiol also shapes reward-related decision-making by shifting choices away from immediately available rewards and toward options requiring greater delay or cognitive control^64,65^.

Traditionally, female gonadal hormone exposure, assessed via menstrual history, has been viewed primarily as a marker of HPG axis activity and reproductive function^66^. However, the present study points to a broader role for gonadal hormone exposure in the regulation of reward circuits, given that longer duration of amenorrhea was linked to diminished reward sensitivity in individuals with LWEDs. These findings may have clinical relevance: transdermal estrogen replacement therapy (in combination with cyclic oral progesterone) could potentially support the normalization of reward functioning in LWEDs with prolonged amenorrhea, paralleling observed estrogen treatment effects in peri- and postmenopausal women^26,27^ and oligo-amenorrheic athletes^36^. However, the therapeutic potential of transdermal estrogen supplementation remains to be evaluated and established in LWED populations. For instance, a randomized, double-masked, placebo-controlled clinical trial of transdermal estrogen replacement (in combination with cyclic oral progesterone) is currently underway at Massachusetts General Hospital to examine estrogen effects on reward processing, including delay discounting and hedonic eating, as well as underlying neural mechanisms in individuals with hypoestrogenic eating disorders (ClinicalTrials.gov identifier: NCT03740204)^67^.

This study was conducted in a moderately large sample of young females with LWEDs and HCs, following a standardized protocol. We accounted for potential confounding by demographically and clinically relevant variables—age, LWED severity via BMI, illness duration, severity of eating disorder symptoms via EDE global score, and caloric intake preceding the hedonic eating task—on the examined associations between menstrual status and reward sensitivity.

Some limitations should be acknowledged. Other clinical variables, for instance psychiatric comorbidities or the use of psychotropic medications, may have influenced the results. They could not be fully accounted for or eliminated from analyses due to their high prevalence in participants with LWEDs. Furthermore, the duration of secondary amenorrhea was based on self-reported menstrual history by recall, which—even though carefully guided by a trained study physician or nurse practitioner—may be less precise than longitudinally maintained menstrual diaries or gonadal hormone levels such as estradiol concentration in blood. However, the former requires a longitudinal study starting from menarche tracking menstrual status over time, and the latter merely captures gonadal status at the time that the gonadal hormone levels were drawn and does not reflect the chronicity of HPG axis suppression. Given our focus on cumulative gonadal hormone deficiency in LWEDs, the total number of missed menstrual cycles since menarche was deemed the most representative metric in our study. Therefore, current-cycle gonadal hormone levels were not evaluated. Moreover, hypothalamic amenorrhea is associated with deficiencies in both estrogen and progesterone. This study does not distinguish between the effects of estrogen, progesterone, or their combined deficiency. For instance, as progesterone levels are also reduced in amenorrheic states^68^, its deficiency may independently or interactively influence neural and behavioral outcomes related to reward processing. However, progesterone has been less extensively studied in individuals with LWEDs; thus, it remains unclear whether progesterone levels are altered at all in this population^69^. In addition to hypogonadism, LWEDs are linked to energy deficiency and associated hormonal alterations, including elevated cortisol and ghrelin levels, and reduced insulin-like growth factor 1 and leptin concentrations^2^. It is possible that some of the associations observed in this study reflect these related hormonal changes. However, because the latter hormonal changes are closely tied to energy status, particularly BMI, we accounted for BMI in our analyses. Another limitation of the present study is that water was available ad libitum. Water intake was not quantified. Potential variability in fluid intake, which can affect satiety and, in consequence, caloric intake during the hedonic eating task, might have occurred. Furthermore, the proportion of daily caloric intake consumed during the mixed-meal breakfast and the snack might impact nutritional state prior to the hedonic eating task. Contrary to expectations, the LWED group reported higher 24-h recall caloric intake than the HC group. Self-reported dietary recalls are inherently vulnerable to reporting bias, particularly potential over-reporting among LWED participants^70^. Moreover, most LWED participants were receiving treatment at the time of study participation (Table 1), and ongoing nutritional rehabilitation may have influenced daily caloric intake. Consequently, 24-h recall data were not considered a reliable proxy for habitual intake or actual energy needs, especially in individuals with restricting type LWEDs. Instead, BMI and absolute caloric intake at the study visit prior to the hedonic eating task were considered more appropriate indicators of nutritional state and energy needs and were therefore incorporated into our main and sensitivity analyses.

Future studies employing disorder-relevant discounting tasks, using food rather than monetary rewards in relation to gonadal hormone exposure in LWEDs, may help to extend the current findings. In addition, larger-scale, long-term longitudinal studies will be important to clarify the relationship between secondary amenorrhea and reward processing across LWED subtypes, as the present study was not designed to and powered for investigating potential distinctions across LWED diagnostic subtypes between menstrual status and reward sensitivity.

## Conclusions

Our findings suggest a potential role of cumulative exposure to gonadal hormones in modulating reward system function in young females with LWEDs, particularly in the domains of disorder-unrelated reward-based decision-making and disorder-specific hedonic eating. These insights may have therapeutic implications, such as enhancing reward-based decision-making and behavior—by targeting features like cognitive overcontrol and anhedonia, which are central to LWED psychopathology^3,23^—through transdermal estrogen replacement therapy, combined with cyclic oral progesterone supplementation. Interventional studies incorporating neuroimaging are warranted to investigate the underlying neural mechanisms and to assess the efficacy of estrogen-based treatments in LWEDs. Additionally, exploring amenorrhea–reward sensitivity associations in other underweight populations, such as individuals with ARFID, will be critical to determine generalizability across the broader LWED spectrum.

## Data Availability

The data that support the findings of this study are not publicly available due to patient privacy and confidentiality requirements. De-identified data may be made available from the corresponding author upon reasonable request and with appropriate institutional and ethical approvals.
